# *Helicobacter pylori* Infection

**DOI:** 10.1155/2015/278308

**Published:** 2015-05-21

**Authors:** Ping-I Hsu, Yoshio Yamaoka, Khean-Lee Goh, Marco Manfredi, Deng-Chyang Wu, Varocha Mahachai

**Affiliations:** ^1^Division of Gastroenterology, Department of Internal Medicine, Kaohsiung Veterans General Hospital and National Yang-Ming University, Kaohsiung, Taiwan; ^2^Environmental and Preventive Medicine, Oita University Faculty of Medicine, Oita, Japan; ^3^University of Malaya Combined Endoscopy Unit, University of Malaya Medical Center, Kuala Lumpur, Malaysia; ^4^Department of Pediatrics, Azienda Ospedaliero Universitaria, University Hospital, Parma, Italy; ^5^Division of Gastroenterology, Department of Internal Medicine, Kaohsiung Medical University Hospital and Kaohsiung Medical University, Kaohsiung, Taiwan; ^6^Gastrointestinal Unit, Chulalongkorn University and Bangkok Medical Center, Bangkok, Pathum Thani, Thailand

*Helicobacter pylori* (*H. pylori*) colonizes the gastric mucosa of more than 50% of the human population. It is the major etiological agent of chronic gastritis, peptic ulcer, gastric mucosa-associated lymphoid tissue lymphoma, and gastric adenocarcinoma. The concomitance of particular genotypes of both pathogen and host may lead to the development of serious gastroduodenal diseases.

With the rising prevalence of antimicrobial resistance, the treatment success of standard triple therapy has recently declined to unacceptable levels in most countries. Several strategies including sequential, concomitant, and hybrid therapies are therefore proposed to increase the eradication rate of first-line treatment for* H. pylori* infection. Since the best first-line eradication regimen with the highest eradication rate and low adverse effects remains unclear and the exact route of transmission is still not exactly known,* H. pylori* infection continues to be a big challenge to all gastroenterologists 30 years after its discovery.

The main focus of the special issue is on recent advances in the diagnosis and treatment of* H. pylori* infection. In addition, the virulence factors and transmission of* H. pylori* are also discussed.

T. Tongtawee et al. in “Correlation between Gastric Mucosal Morphologic Patterns and Histopathological Severity of* Helicobacter pylori* Associated Gastritis Using Conventional Narrow Band Imaging Gastroscopy” investigated specific gastric mucosal morphologic patterns of* Helicobacter pylori* gastritis by NBI. The data indicate that mucosal morphologic patterns of* H. pylori* gastritis can be reliably identified using C-NBI gastroscopy with good correlation with inflammation grading.

W.-C. Tai et al. in “Seven-Day Nonbismuth Containing Quadruple Therapy Could Achieve a Grade “A” Success Rate for First-Line* Helicobacter pylori* Eradication” conducted a prospective trial to compare the efficacies of nonbismuth containing quadruple therapy and standard triple therapy in Taiwan. The results showed that a 7-day nonbismuth containing quadruple therapy achieved a higher eradication rate than 7-day standard triple therapy (95.6% versus 79.3% by per-protocol analysis). In the paper entitled “Levofloxacin-Amoxicillin/Clavulanate-Rabeprazole versusa Standard Seven-Day Triple Therapy for Eradication of* Helicobacter pylori* Infection,” M.-C. Chen et al. demonstrated that a seven-day regimen containing levofloxacin, amoxicillin/clavulanate, and rabeprazole was superior to a standard triple regimen containing clarithromycin, amoxicillin, and rabeprazole in Taiwan.

Regarding the second-line therapy, G.-H. Jheng et al. in “Comparison of Second-Line Quadruple Therapies with or without Bismuth for* Helicobacter pylori* Infection” conducted a randomized controlled trial to compare the efficacies of standard quadruple regimen (rabeprazole, bismuth subcitrate, tetracycline, and metronidazole) and a modified concomitant regimen (rabeprazole, amoxicillin, tetracycline, and metronidazole) after failure of standard triple therapy. Intention-to-treat analysis showed that the two rescue quadruple therapies had comparable eradication rates (91.9% and 89.7%, resp.). The results suggest that the 10-day modified concomitant regimen can be an alternative rescue therapy for* H. pylori* infection in bismuth-unavailable countries. In the paper entitled “Quinolone-Containing Therapies in the Eradication of* Helicobacter pylori,*” S.-K. Chuah et al. review the efficacies of quinolone-containing regimens for* H. pylori* infection and discuss the public health issue of emerging resistant strains of mycobacteria following quinolone-containing* H. pylori* eradication therapy.

In the paper entitled “The Prevalence of* Helicobacter pylori* Virulence Factors in Bhutan, Vietnam, and Myanmar Is Related to Gastric Cancer Incidence,” T. T. H. Trang et al. examined the status of* cagA*,* vacA*,* jhp0562, *and*β-(1,3)galT* in* H. pylori*-infected patients from Bhutan, Vietnam, and Myanmar. The data suggest that the* cagA*,* vacA *s1,* vacA *m1, and* jhp0562*-positive/*β-(1,3)galT*-negative may play a role in the development of gastric cancer.

Biofilm formation is critical not only for environmental survival but also for successful infection. H. Yonezawa et al. in “Biofilm Formation by* Helicobacter pylori* and Its Involvement for Antibiotic Resistance” demonstrated that biofilm formation of* H. pylori* could decrease susceptibility to antibiotics and* H. Pylori* antibiotic resistance mutations were more frequently generated in biofilms than in planktonic cells.

In the paper entitled “Helicobacteraceaein Bulk Tank Milk of Dairy Herds from Northern Italy,” V. Bianchini et al. revealed that* H. pylori* was not identified in any of the samples from the bulk tank milk of dairy cattle herds. The data suggest that, at least in the farming conditions of the investigated area, bovine milk does not represent a potential source of infection.

*Ping-I Hsu* *Yoshio Yamaoka* *Khean-Lee Goh* *Marco Manfredi* *Deng-Chyang Wu* *Varocha Mahachai*

